# GLP-1 Agonist As Adjunct To Antidepressants In Patients With Major Depressive Disorder: A Randomized, Double-Blind, Placebo-Controlled Trial.

**DOI:** 10.1192/j.eurpsy.2026.11401

**Published:** 2026-07-31

**Authors:** A. N. R. B. Saad

**Affiliations:** Neuropsychiatry, Menoufia University, Menoufia, Egypt

## Abstract

**Introduction:**

Glucagon-like peptide-1 (GLP-1) receptor agonists (GLP-1 RAs), initially known for their ability to help manage diabetes, these drugs are now showing promise as a fresh approach to treating mental health conditions especially Major depressive disorder (MDD), as it exerts antiinflammatory effect which has antidepressant effect.

**Objectives:**

I aimed to investigate its safety and efficacy liraglutide as GLP-1 agonist in patients with MDD and its effect on inflammatory profile of the patients by conducting double- blind, randomized, placebo- controlled study.

**Methods:**

120 participants with MDD (DSM- V criteria) with body mass index (BMI) ≥ 27 kg/m^2^ enrolled and Hamilton Depression Rating Scale (HDRS) score >20 were treated with liraglutide 3.0 mg/day or placebo plus escitalopram 20 mg once daily for six weeks. Patients were evaluated by HDRS scores (weeks 0, 2, 4, and 6). Serum levels of CREB1, BDNF, TNF– α, NF- κB, and FAM19A5 were assessed pre- and post- treatment.

**Results:**

Participants (*n* = 120) were 41.2 ± 12.6 years; BMI = 33.9 ± 11.8 kg/m^2^; 60% women. Co- administration of liraglutide had markedly decreased HDRS score at all- time points compared to the placebo group (p < 0.001). Early improvement, response, and remission rates after 6 weeks were significantly higher in the GLP-1 group (70%, 80%, 90%, respectively) than in the placebo group (25%, 65%, 50% respectively) (p < 0.001). Moreover, the GLP-1 group was superior to the placebo group in modulation of the measured neurotrophic and inflammatory biomarkers. Percent weight loss was significantly greater in the liraglutide versus the placebo group (3.2 ± 1.0% vs. 0.2 ± 0.1%, *p* = 0.005).

**Conclusions:**

Glp-1 agonist can be used as adjunct treatment for patients with MDD and counteract the metabolic side effects of antridepressants

**Disclosure of Interest:**

None Declared

